# *In Silico* Epitope Prediction Analyses Highlight the Potential for Distracting Antigen Immunodominance with Allogeneic Cancer Vaccines

**DOI:** 10.1158/2767-9764.CRC-21-0029

**Published:** 2021-11-30

**Authors:** C. Alston James, Peter Ronning, Darren Cullinan, Kelsy C. Cotto, Erica K. Barnell, Katie M. Campbell, Zachary L. Skidmore, Dominic E. Sanford, S. Peter Goedegebuure, William E. Gillanders, Obi L. Griffith, William G. Hawkins, Malachi Griffith

**Affiliations:** 1 Department of Surgery, Washington University School of Medicine, St. Louis, Missouri.; 2 Department of Medicine, Washington University School of Medicine, St. Louis, Missouri.; 3 McDonnell Genome Institute, Washington University School of Medicine, St. Louis, Missouri.; 4 Siteman Cancer Center, Washington University School of Medicine, St. Louis, Missouri.; 5 Department of Genetics, Washington University School of Medicine, St. Louis, Missouri.

## Abstract

Allogeneic cancer vaccines are designed to induce antitumor immune responses with the goal of impacting tumor growth. Typical allogeneic cancer vaccines are produced by expansion of established cancer cell lines, transfection with vectors encoding immunostimulatory cytokines, and lethal irradiation. More than 100 clinical trials have investigated the clinical benefit of allogeneic cancer vaccines in various cancer types. Results show limited therapeutic benefit in clinical trials and currently there are no FDA-approved allogeneic cancer vaccines. We used recently developed bioinformatics tools including the pVACseq suite of software tools to analyze DNA/RNA-sequencing data from the The Cancer Genome Atlas to examine the repertoire of antigens presented by a typical allogeneic cancer vaccine, and to simulate allogeneic cancer vaccine clinical trials. Specifically, for each simulated clinical trial, we modeled the repertoire of antigens presented by allogeneic cancer vaccines consisting of three hypothetical cancer cell lines to 30 patients with the same cancer type. Simulations were repeated ten times for each cancer type. Each tumor sample in the vaccine and the vaccine recipient was subjected to human leukocyte antigen (HLA) typing, differential expression analyses for tumor-associated antigens (TAA), germline variant calling, and neoantigen prediction. These analyses provided a robust, quantitative comparison between potentially beneficial TAAs and neoantigens versus distracting antigens present in the allogeneic cancer vaccines. We observe that distracting antigens greatly outnumber shared TAAs and neoantigens, providing one potential explanation for the lack of observed responses to allogeneic cancer vaccines. This analysis provides additional rationale for the redirection of efforts toward a personalized cancer vaccine approach.

**Significance:**

A comprehensive examination of allogeneic cancer vaccine antigen repertoire using large-scale genomics datasets highlights the large number of distracting antigens and argues for more personalized approaches to immunotherapy that leverage recent strategies in tumor antigen identification.

## Introduction

Cancer vaccines were one of the first immunotherapies to be tested in humans (1) but progress has been limited. By augmenting a patient's immune response to antigens expressed (or overexpressed) by malignant cells, cancer vaccines are designed to provide systemic antitumor immunity while limiting the off-site toxicities associated with chemotherapy, radiotherapy, and nonspecific immunotherapies such as immune checkpoint inhibition (ICI). Initial cancer vaccines predominantly targeted proteins expressed at higher levels in tumors compared with normal tissue, so-called tumor-associated antigens (TAA), such as gp100 in melanoma or mesothelin in pancreatic cancer (2, 3). Early iterations were often peptide-based, targeting one or more previously defined TAA (4). While capable of generating an antigen-specific response (5, 6), overall clinical response rates to these vaccines were very low (7) and immune responses did not reliably correlate with improved outcome (6, 8). One hypothesis for their failure is that the immune response to an antigen is influenced by the context in which it is presented. To generate a more robust response, investigators developed allogeneic cancer vaccines, human cancer cell lines in whole-cell or cell lysate form (Supplementary Fig. S1A and S1B). Allogeneic cancer vaccines carried the added potential to target antigens not yet identified.

Allogeneic cancer vaccines were first used in advanced melanoma (1) and have since been tested in lung (9), sarcoma (10), neuroblastoma (11), prostate (12), renal cell (13), breast (14), colon (15), and pancreatic cancer (16)*.* Allogeneic cancer vaccines provided a safe platform for the simultaneous delivery of multiple known and unknown TAAs, and enthusiasm for this approach translated into more than 100 phase I/II clinical trials between 1988 and 2020 (Supplementary Table S1). However, only a fraction of these progressed to a phase III trial (Table 1), and none demonstrated a survival benefit. Nevertheless, allogeneic cancer vaccines continue to be explored with ten new phase I/II trials initiated since 2016 (Supplementary Table S1), including one in 2020.

**TABLE 1:** Summary of phase III clinical trials involving allogeneic cancer vaccines.

Cancer type	Number of trials (year began)	Enrollment (*n*)	Outcome measured	Result	Vaccine (cell lines)	Added agents
Melanoma	7 (1987, 1992, 1996, 1997, 1998^a^, 1998^a^, 2009^b^)	1417	DFI, Median OS, 5yr DFS	NS	Vaccinia melanoma Oncolysate, Canvaxin (M101, M24, M10), Melacine, CSF470	Vaccinia virus, BCG, IFN-a2b, rhGM-CSF
NSCLC	2 (2008, 2013^a^)	667	OS	NS	Belagenpumatucel-L (SK-LU1/HBA2, H520/HBA2, RH2/HBA2, H460/HBAA/2) Tergenpumatucel-L	TGF-B2 antisense modified, murine alpha-1, 3-gal modified Cell line
Prostate adenocarcinoma	2 (2004^a^, 2005^a^)	626	OS	NS	GVAX (PC3, LNCaP)	GM-CSF
Pancreatic adenocarcinoma	2 (2010, 2013^a^)	1024	OS	NS	Algenpantucel-L	Murine alpha-1,3-gal modified cell line

Abbreviations: DFI, disease-free interval; DFS, disease-free survival; NS, “not significant” difference between groups; OS, overall survival.

^a^Indicates trial terminated early.

^b^Indicates trial without published data.

While several theories have been proposed to explain the lack of success of allogeneic cancer vaccines (17, 18), we hypothesized that at least one important contributor to their lack of efficacy is the relative paucity of beneficial antigens provided by these vaccines compared with the number of distracting antigens. Beneficial tumor antigens exist in three broad categories: overexpressed self-antigens in the form of TAAs, antigens resulting from somatic mutations present in the cancer (i.e., neoantigens), and viral antigens in the case of viral-induced tumors (e.g., HPV). For the purposes of the cancer types explored here, we focus our analysis on TAAs and neoantigens. The recently developed pVACtools software suite (19) provided the computational framework to rigorously explore the antigenic repertoire supplied by allogeneic cancer vaccines. Herein, we utilize the TCGA database to simulate allogeneic cancer vaccine clinical trials in an effort to quantify the ratio of beneficial antigens to distracting antigens within cancer types. The extent to which beneficial antigens are outnumbered by off-target antigens present in allogeneic vaccines has not been previously explored, and our analysis illustrates a potential shortcoming of this approach.

## Materials and Methods

### Simulation of Allogeneic Tumor Cell Vaccine Clinical Trials

**FIGURE 1:**
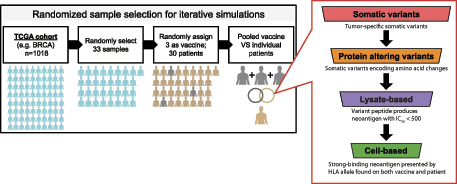
Simulated clinical trial work flow and shared neoantigen analysis. Using cancer-type matched cohorts from the TCGA, 33 samples were selected randomly and designated as either “vaccine” (*n* = 3) or “patient” (*n* = 30). Analysis was then performed to determine the number of shared tumor-specific neoantigens between the pooled vaccine and each patient individually. Right, a neoantigen analysis workflow is depicted. Tumor-specific neoantigens were determined for each sample individually in the following order. All somatic variants in the tumor were first identified. Non–protein-altering variants were excluded. The total pool of possible peptides was then calculated, and those that did not result in a predicted neoantigen with an IC_50_ < 500 nm were excluded for the lysate-based analysis. In addition, predicted neoantigens not presented on both the vaccine and patient's MHC haplotype were excluded for the cell-based analysis (Materials and Methods).

A series of hypothetical clinical trials were simulated using samples from The Cancer Genome Atlas (TCGA; ref. 20) to represent both the somatic and germline variant landscape of individual patients and the complexity of allogeneic cancer vaccines created from multiple tumor cell sources. For each clinical trial simulation, 33 tumor samples were randomly selected, whereby three samples were designated as the allogeneic cancer vaccine cocktail and the remaining 30 samples were designated as hypothetical patients to assess overlap of antigens presented by the vaccine with the repertoire of antigens presented by the tumor (Fig. 1). This simulation was repeated 10 times each for 30 different tumor subtypes. For each tumor type, for each simulation, tumor sampling was performed without replacement using the full set of individuals available for that tumor type. Overlap of tumor identity between iterations was possible. The assignment of tumors to the vaccine cocktail or patient group was also random.

### HLA Typing

Human leukocyte antigen (HLA)-I/II haplotypes were obtained for TCGA samples from the Cancer Immune Atlas (TCIA) via a controlled access data use agreement for the TCGA managed by dbGaP (21). The TCIA did not have HLA types for all TCGA cases with somatic variants available in the Genomic Data Commons (GDC). If samples had previously performed HLA typing from the TCIA, these calls were repurposed for this analysis. If samples did not have previously performed HLA typing, OptiType (22) was used to predict HLA types. Briefly, for these samples, raw whole-exome data was downloaded from the GDC and used as input for OptiType.

### TAA Analysis

TAA analysis was limited to established TAA genes deemed relevant to each cancer type studied. Gene names and reference transcripts for these targets were as follows: gp100 (also known as PMEL; ENST00000548747.5), CEA (ENST00000598976.1), HER2 (also known as ERBB2; ENST00000269571.10), Ny-ESO-1 (CTAG1B; ENST00000328435.2), MUC1 (ENST00000338684.9), Mesothelin (also known as MSLN; ENST00000545450.7), PSA (also known as KLK3; ENST00000326003.2), PAP (also known as ACP3; ENST00000336375.10), MART-1 (also known as MLANA; ENST00000381477.8), and VEGFR1 (also known as FLT1; ENST00000282397.9). Protein sequences for these reference transcripts were obtained using Ensembl BioMART (23). Candidate TAA peptides encoded by each full-length protein sequence were identified using the pVACbind module of pVACtools. Every possible 9 amino acid peptide from these full-length protein sequences was analyzed for binding against every HLA class I allele observed across all patient and vaccine samples examined. Candidate TAA peptides were defined as those having a predicted peptide HLA I binding affinity < 500 nm (median of multiple binding prediction algorithms as described above for the neoantigen analysis) for the patient. If multiple adjacent 9-mer peptides extracted from the full-length sequence were considered strong HLA binders and their overlapping peptide sequence was > 44% (4 of 9 amino acids), only one of these overlapping candidates was counted. For the purpose of this analysis, all genes were assumed to be robustly expressed in the vaccine samples (as would likely be the case in the sample chosen for a real allogeneic vaccine). In each hypothetical patient sample (TCGA cases), expression data were obtained from the GDC and a TAA gene was considered expressed if it was expressed above the 50th percentile of all nonzero (FPKM) genes within that tumor. Each peptide candidate was only counted if the TAA gene was expressed by the patient. Analysis was performed to reflect two alternate vaccine models: a “lysate-based” vaccine model and a “cell-based” vaccine model. Shared TAAs in the “lysate-based” analysis represent strong binding (IC_50_ < 500 nm) TAAs expressed in the patient, as described above, with no additional requirements. Under the “cell-based” analysis, a TAA peptide had to be a strong binding candidate for at least one HLA allele shared by the cells of the patient and the cells of the vaccine samples. This analysis takes into account the scenario where intact allogeneic cancer cells are used in vaccination and are responsible for presenting antigens directly to the patient's immune system. As a result, shared TAAs identified in the cell-based analysis represent a subset of those identified by lysate analysis (requiring sharing of both peptides and HLA haplotypes between vaccine and patient). While the predominant mechanism is likely cross-presentation of antigens in apoptotic bodies from the lysed cell (i.e., lysate-based analysis; ref. 24), we include cell-based analysis for the sake of completeness.

### Somatic Variant Calling/Filtering/Annotation

For analysis of the neoantigen repertoire, we first identified somatic variants present in individual cancers. Somatic variants for TCGA samples were obtained from the Genomic Data Commons (GDC) Portal using the GDC Data Transfer Tool (25). Somatic variant files used were those generated with the GDC Mutect2 workflow (26). VCF files were decompressed with gunzip and recompressed and indexed with bgzip and Tabix (27). All variants marked as failing any filter of the GDC Mutect2 workflow were removed using vcftools with options “–recode” and “–remove-filtered-all” (28). Variants called by MuTect2 were further filtered to require tumor DNA variant allele fraction >5% and tumor depth >5 reads. Somatic variants were annotated for their transcriptional consequence using the Ensembl Variant Effect Predictor (VEP) version 93.2 using default parameters with the following modifications to facilitate neoantigen analysis in the following sections: “–plugin Downstream –plugin Wildtype –symbol –tsl” (29). A tabular variant report was generated from each VCF file using the GATK module VariantsToTable to extract basic variant information: CHROM, POS, REF, and ALT (30). The variant report was then supplemented with transcript effect information using the VAtools “VEP Annotation Reporter” module (http://vatools.org/) to extract the following fields: Feature, Consequence, SYMBOL, Protein_position, and Amino_acids. The annotated VCF file was used for neoantigen identification using pVACtools (19, 31) as described below and the tabular report was used to compare somatic variants between simulated allogeneic vaccines and patients.

### Neoantigen Prediction

Using HLA typing results and filtered variants obtained above, neoantigen prediction was performed using the pVACseq package of pVACtools (v.4.1) (19, 31). Briefly, ten different epitope/HLA binding affinity algorithms were employed to compile a list of putative neoantigens that have a varying level of predicted efficacy: NNalign, NetMHC, MHCFlurry, NetMHCcons, NetMHCpan, PickPocket, SMM, SMMPMBEC, MHCnuggets, and SMMalign. Parameters for pVACseq included limiting predictions for frameshift mutations to less than 500 amino acids downstream of the variant. Neoantigens selected for further analysis were required to have < 500 nm predicted binding affinity to be considered a candidate neoantigen (using the median binding affinity of all algorithms that could be applied to each particular peptide:HLA pair; Fig. 1). When a single somatic variant results in multiple peptide registers (e.g., same mutant amino acid at different positions in a 9-mer) or multiple peptide lengths that are predicted to be strong binders to HLA, these were not double counted. In other words, each neoantigen count refers to at least one strong binding peptide arising from a somatic mutation.

### Comparison of Vaccine Neoantigen Predictions Against Simulated Patient Predictions

Somatic variants for each of the three samples used in the simulated vaccine were merged into a single list (Fig. 1). Overlap of somatic variants between the simulated vaccine and individual patients required a direct match with regard to the altered amino acid sequence. Analysis was performed to reflect two alternate vaccine models: a “lysate-based” vaccine model and a “cell-based” vaccine model as described above for the case of shared TAAs. Briefly, a neoantigen was considered to be shared in the “lysate-based” analysis if an overlapping somatic variant produced a strong binding (IC_50_ < 500 nm) neoantigen in an individual patient. Under the “cell-based” analysis, a neoantigen was considered to be shared if an overlapping somatic variant produced a strong binding (IC_50_ < 500 nm) neoantigen in both the vaccine and an individual patient.

### Germline Polymorphism Calling/Filtering/Annotation

Germline variants were called using GATK's HaplotypeCaller using the default parameters (30). Single-nucleotide polymorphisms (SNP) were further filtered using GATK's VariantFiltration and filtering parameters of QD < 2.0, FS > 60.0, MQ < 40.0, and SOR > 3.0. Small insertions and deletions (indels) were further filtered using GATK's VariantFiltration and filtering parameters of QD < 2.0, FS > 200.0, MQ < 40.0, and SOR > 5.0.

### Germline Alloantigen Analysis

Estimation of potential off-target alloantigens present in allogeneic vaccines relied on germline variant analyses described in the previous methods section. In each patient sample, the genotype of all SNPs and small insertions and deletions was considered and compared with the genotype of such polymorphisms in the sample of each hypothetical allogeneic vaccine. Potential alloantigen producing germline polymorphisms were those present in at least one sample of the allogeneic vaccine but absent from (and therefore potentially foreign to) the patient sample. This evaluation was performed for every patient in each of 10 simulated trials for five cancer types (total of 30 patients × 5 cancer types × 10 random trial iterations = 1,500 patients). Each of these comparisons yielded thousands of potential variants to test for HLA I binding. The altered amino acid sequence arising from each potentially foreign variant needed to be tested against all HLA alleles of the either the corresponding patient (lysate-based model) or both the patient and vaccine (cell-based model) samples. Because the foreign variants represented common polymorphisms, some combinations of candidate variant and HLA alleles were observed in multiple patients and clinical trial simulations. For computational efficiency, a database of germline variant HLA pairs to be tested was created. Each pair was subjected to antigen analysis using the pVACseq module of pVACtools using the same ten binding prediction algorithms as described above for the neoantigen analysis. In contrast to the neoantigen analysis where such calculations were only performed for the relatively rare instances of somatic variants being shared between vaccines and patients, this analysis of foreign germline variants involved orders of magnitude more binding predictions. Antigen binding predictions were required for 6,559,512 variant–HLA pairs. Analysis with pVACseq required approximately 10 days of computation on a high-performance compute cluster using 500 nodes with 4 CPUs each. Antigen candidates were identified from these results essentially as described for the neoantigen and TAA analyses as described above. Summarization of alloantigen peptide counts for each patient was again considered under the “lysate-based” and “cell-based” vaccine assumptions as described above. Under the lysate-based assumption, to be counted, a peptide had to be a strong HLA-binding candidate for at least one HLA allele of the patient's HLA type (i.e., presentable by the patient's cells). Under the “cell-based” assumption, a peptide had only to be a strong binding candidate for at least one HLA allele of the cells of the vaccine samples.

### Data Availability

Exome data from cases representing each cancer type were obtained from TCGA (20) and downloaded via the Genomic Data Commons (32). This data can be accessed under dbGaP study accession phs000178.

## Results

### TAAs are Modestly Expressed Throughout Cancer Types

**FIGURE 2:**
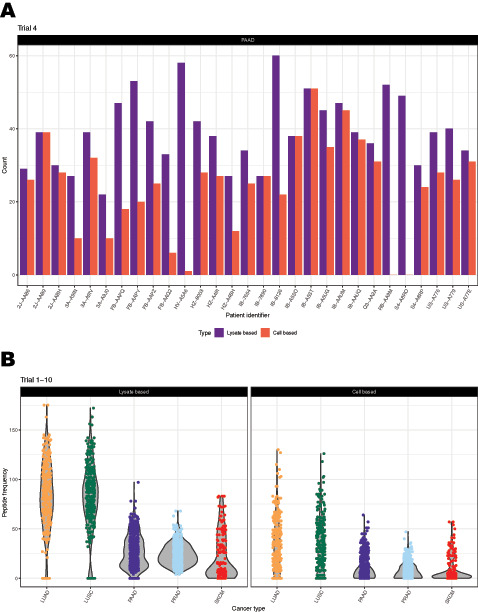
TAA expression analysis. **A,** TAA expression in a single simulation of a pancreatic adenocarcinoma clinical trial. Tumor associated antigen counts (MUC1 and Mesothelin for PAAD) are summarized for an example trial simulation in pancreatic adenocarcinoma. TAAs were assessed for each patient after taking into account the MHC alleles for either patient only (lysate-based analysis) or patient and vaccine (cell-based analysis), requiring evidence for both presentation of TAA peptide and robust expression of the TAA gene (Methods). **B,** TAA expression in each of five cancer types. For each cancer type, select established TAA genes were considered (Gp100, Ny-ESO-1 and MART-1 for SKCM; VEGFR1 and Ny-ESO-1 for LUAD; VEGFR1 and Ny-ESO-1 for LUSC; PSA and PAP for PRAD). Counts of expressed TAAs across 300 patients for each tumor type are shown as violin plots with individual points representing the count for each simulated patient overlaid.

Expression of commonly identified TAAs across patients within a cancer type was assessed as a surrogate for the potential antitumor effect provided by these vaccines. Established TAAs were selected for each of the five cancer types commonly seen in allogeneic vaccine clinical trials: Gp100, Ny-ESO-1, and MART-1 for melanoma (SKCM); VEGFR1 and Ny-ESO-1 for lung adenocarcinoma (LUAD); VEGFR1 and Ny-ESO-1 for lung squamous carcinoma (LUSC); PSA and PAP for prostate adenocarcinoma (PRAD); and MUC1 and Mesothelin for pancreatic adenocarcinoma (PAAD). For each cancer type, clinical trials were simulated (see Materials and Methods). After considering HLA alleles for patient and vaccine, each patient was evaluated for presentation of the TAA peptide and robustness of their TAA expression using RNA data (Methods). A single clinical trial iteration in PAAD is shown for illustration, presenting both “lysate-based” and “cell-based” analysis (Fig. 2A). Here, lysate-based analysis represents TAAs expressed in the patient and predicted to be strong binders to at least one of the patient's HLA alleles,whereas cell-based analysis represents TAAs expressed by the patient and predicted to be strong binders to at least one HLA allele shared by the patient and vaccine. By lysate-based analysis, 83.3% of patients expressed between 25 and 50 TAAs (MUC1, mesothelin), whereas 16.7% exhibited between 50 and 60. This process was repeated for a total of 10 clinical trial simulations, to simulate the treatment of 300 patients across five different cancer types (Fig. 2B). LUAD and LUSC had the most robust expression of TAAs (VEGFR1 and Ny-ESO-1) among the five cancer types examined. Patients shared between 25 and 155 TAAs with the vaccine by lysate-based analysis, with approximately 30% of patients exhibiting greater than 100 shared antigens. However, PAAD, PRAD (PSA, PAP), and SKCM (Gp100, Ny-ESO-1, and MART-1) exhibited significantly fewer TAAs across their respective patient populations. Patients ranged between 25 and 75 shared antigens by lysate-based analysis, with 7% of SKCM patients and 3.6% of PRAD patients expressing more than than 50 TAAs. TAA expression was consistent throughout patients within a cancer type; however, the level of expression varied widely by cancer type.

### Allogeneic Cancer Vaccines Share Exceptionally Few Shared Neoantigens with Individual Patients

**FIGURE 3:**
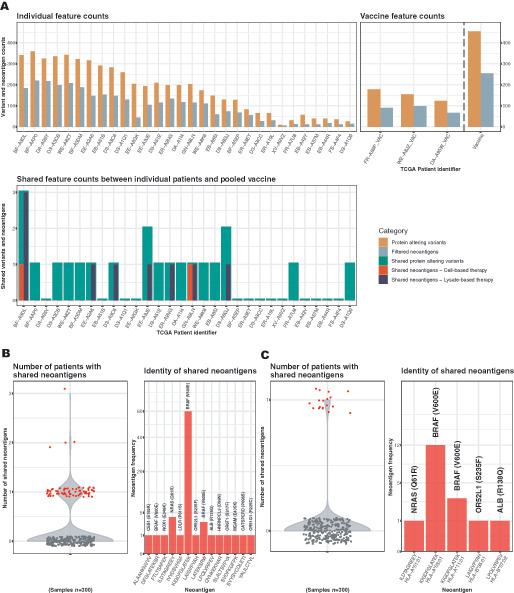
Shared neoantigen analysis in melanoma. **A,** Single Iteration of a simulated clinical trial using TCGA's melanoma (SKCM) cohort. The count of each patient's (*n* = 30) protein-altering somatic variants and predicted neoantigens are shown in the top left panel. Quantification of each designated “vaccine” sample, both individually (*n* = 3) and as a pooled “Vaccine” are provided in the top right panel. Shared variants and predicted neoantigens by cell-based and lysate-based analysis are shown in the bottom panel. **B,** Lysate-based neoantigen analysis of melanoma simulated clinical trial. Ten clinical trials in melanoma were simulated analyzing a total of 300 patients. The total number of shared neoantigens that each patient shared with the vaccine, by lysate-based analysis, is shown in the left. In patients who had one or more shared neoantigens with the vaccine, the identity and frequency of each neoantigen is shown in the right. **C,** Cell-based neoantigen analysis of melanoma simulated clinical trial. Ten clinical trials in melanoma were simulated analyzing a total of 300 patients. The total number of shared neoantigens that each patient shared with the vaccine, by cell-based analysis, is shown in the left. In patients who had one or more shared neoantigens with the vaccine, the identity and frequency of each neoantigen is shown in the right.

While the immunologic benefits from allogeneic vaccines were believed to stem predominantly from high expression of TAAs, somatic mutations (i.e., neoantigens) shared between the patient and vaccine may represent an additional source of beneficial antigens provided by this approach. At their inception, the frequency of shared neoantigens found in allogeneic vaccines was not expected to be sufficiently high to be immunologically relevant (33), although this matter has not been explored in the literature. Therefore, all simulated clinical trials were assessed to determine the frequency of shared neoantigens among treated patients. A single clinical trial iteration in melanoma (SKCM) is shown for illustration (Fig. 3A). As outlined above, a pooled vaccine was compared with 30 individual patients – shared protein altering variants (PAV) and neoantigens from lysate-based or cell-based analysis are shown. The pooled vaccine represented a total of 455 unique PAVs and 254 filtered neoantigens. Individual patients averaged 177 PAVs and 143 filtered neoantigens. 17 (56.6%) patients had at least a single overlapping PAVs with the vaccine. By lysate-based analysis, seven (23.3%) patients shared a single neoantigen with the vaccine, while cell-based analysis showed that only two patients (6.6%) shared a single neoantigen with the vaccine. This process was repeated for a total of 10 clinical trial iterations, simulating 300 patients treated with 10 distinct simulated vaccines. Lysate-based analysis (Fig. 3B) and cell-based analysis (Fig. 3C) for 10 iterations of SKCM are shown. As explained above, cell-based overlaps are, by definition, a subset of shared neoantigens identified in lysate-based analysis. In total, 73 (24.3%) patients shared at least one neoantigen with the vaccine by lysate-based analysis. One patient had three shared neoantigens, three patients had two shared neoantigens, and 69 patients had a single overlapping neoantigen. Of the 73 patients with a shared neoantigen, 64 (87.7%) were related to a *BRAF* mutation. By the more restrictive cell-based analysis, only 18 (6%) patients shared any neoantigen with the vaccine, 15 of which were *BRAF* mutations.

**FIGURE 4:**
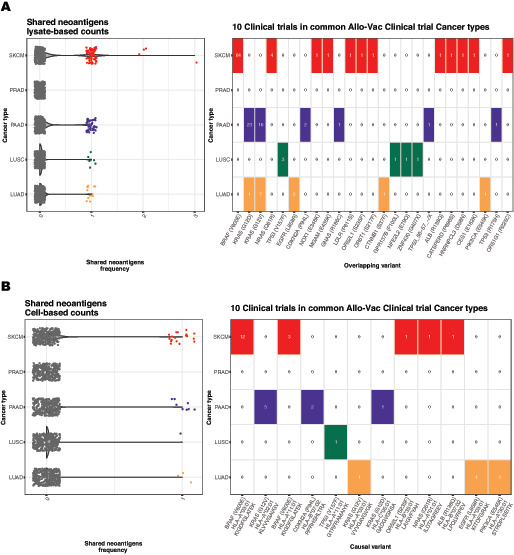
Shared neoantigen analysis in common allogeneic vaccine cancer types. **A,** Lysate-based overlap analysis. Counts of shared neoantigens per patient for each cancer type based on a lysate-based vaccine administration are shown on the left. Counts summarize 10 complete trial simulations (300 simulated patients total) for each cancer type. A heatmap of shared neoantigen identities from combined trials in each cancer type (300 simulated patients) is shown on the right. **B,** Cell-based overlap analysis. Counts of shared neoantigens per patient for each cancer type based on cell-based vaccine administration are shown on the left. Counts summarize 10 complete trial simulations (300 simulated patients) for each cancer type are shown on the left. A heatmap of shared neoantigen identities from combined trials in each cancer type (300 simulated patients) is shown on the right.

10 such iterations of vaccine trials were performed in all 30 cancer types available on TCGA that yielded comparable results (Supplementary Figs. S2 and S3). In addition to melanoma (SKCM), the four other cancer types most commonly associated with allogeneic vaccines (PRAD, PAAD, LUAD, LUSC) are shown in Fig. 4A and B (lysate- and cell-based analysis, respectively). PAAD had the second highest number of patients with any overlap with the vaccine behind SKCM. Forty-four (14.6%) patients had a single shared neoantigen by lysate-based analysis, 39 of which represented *KRAS* mutations (G12D or G12V). By cell-based analysis, 8 patients had shared neoantigens, 6 of which were KRAS related. LUAD had the third highest number of shared neoantigens with 12 patients (4%), 8 of which represented *KRAS* mutations (G12D or G12V). In the cell-based analysis, 3 patients had overlapping neoantigens, 1 of which was KRAS related. LUSC had very few patients with overlapping neoantigens, 6 patients (2%) with a single overlapping neoantigen by lysate-based and only one patient by cell-based analyses. In 300 PRAD patients, none shared a single neoantigen with the vaccine pool by lysate- or cell-based analysis.

### Allogeneic Vaccines Represent a Major Source of Off-Target Alloantigens

**FIGURE 5:**
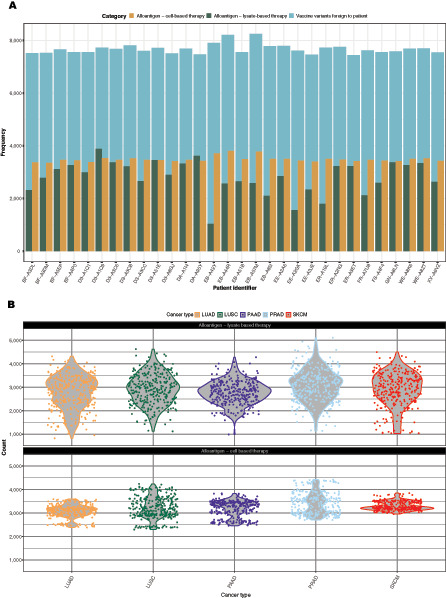
Genome-wide germline variant and alloantigen analysis. **A,** Alloantigen analysis in a single simulation of a melanoma clinical trial. Nonspecific alloantigen presentation provided by allogeneic vaccines are quantified for each of 30 patients. Unique protein altering variants (teal) are shown along with those predicted to be strong binding (IC_50_ < 500 nm) by cell based (tan) and lysate-based (green) analysis. **B,** Alloantigen analysis for five common cancer types, lysate and cell-based. The counts of predicted alloantigens corresponding to 10 simulated vaccine trials for five cancer types (300 simulated patients per cancer type) are depicted as violin plots for both a lysate-based (top) and cell-based (bottom) analysis.

While TAA's appear to be modestly expressed across most patients, these are being presented alongside off-target “alloantigens”. These include either unique antigens resulting from naturally occurring polymorphisms in a population, or normal protein antigens being presented on novel donor HLA alleles (in the case of a cell-based analysis assuming direct presentation). Because allogeneic HLA alleles and protein-altering germline polymorphisms are novel to the patient, they carry the potential for nonspecific immunogenicity. Evidence that these alloantigens are recognized by the immune system and are immunogenic can be found in solid organ transplantation, where increased frequency of alloantigens are believed to be responsible for higher rates of graft rejection (34) and part of the original rationale for allogeneic cancer vaccines. A single iteration of the SKCM simulated trial is shown (Fig. 5A). The vaccine consistently presents between 7,000 and 8,000 alloantigens, approximately two orders of magnitude larger than the beneficial antigens quantified above. Of these, between 2,000 and 4,000 were predicted to be strong binding antigens, with IC_50_ < 500 nm. This finding was consistent across the five cancer types most commonly seen in allogeneic vaccine trials, and for both lysate- and cell-based analysis (Fig. 5B).

## Discussion

The objective of this work was to investigate the antigen repertoire associated with allogeneic cancer vaccines. Our work uses innovative bioinformatics tools to provide a novel quantitative evaluation of the allogeneic vaccine antigen repertoire, illustrating the extent to which beneficial TAAs and neoantigens are vastly outnumbered by distracting alloantigens. These data suggest one potential limitation of the allogeneic cancer vaccine platform and rationale for the lack of success seen in these approaches at the phase III level.

Here we confirm the hypothesis that very few shared neoantigens are present in allogeneic vaccines. In melanoma, a cancer with a well-documented high somatic mutation prevalence (35), only 73 patients (24.3%) have any overlapping neoantigens with a simulated allogeneic vaccine, and of these, the majority (69 of 73, 88%) have only a single neoantigen in common. In addition, the majority of shared neoantigens observed are *BRAF* mutations (64 of 78, 82%), a known driver mutation for melanoma and previously documented in as many as 66% of all malignant melanoma cases (36). Not only is the presence of shared *BRAF* mutations in our analysis to be expected, but it is also a mutation for which an FDA-approved targeted therapy already exists (37). Excluding these patients for whom BRAF-directed therapy would be indicated, only 14 of the 300 (4.6%) patients will benefit from a vaccine-provided neoantigen. A similar observation is made in PAAD and LUAD, for whom the overwhelming majority of shared neoantigens were *KRAS* mutations – 90% and 92%, respectively. *KRAS* is a known driver mutation for PAAD but has proven elusive as a therapeutic target (38). KRAS-targeted therapy has also been defined by failure in LUAD, apart from a recent breakthrough in the form of Sotorasib (39, 40). When excluding these expected driver mutations described above, there are exceedingly few patients (1.7% and 1.3% in PAAD and LUAD, respectively), who have even a single overlapping neoantigen with the simulated vaccine. In cancers without classical driver mutations, even fewer overlapping neoantigens were observed – 0% and 2% of patients with PRAD and LUSC, respectively, had any overlap with the vaccine. These data suggest that an immunologic benefit from allogeneic vaccines would rely to a much greater extent on TAA expression and not shared neoantigens.

Indeed, we observed TAA expression consistently across five different tumor types that could provide an immunologic benefit as a result. However, attempts to vaccinate against TAAs have yielded minimal success since their inception in the 1980s (41). This is likely due to multiple factors—TAAs, as self-antigens, are potentially subject to central and acquired tolerance that would restrain the resulting T-cell response (42). Another consequence of vaccinating against self-antigens is the potential for an autoimmune response, although this is rare and likely depends on the type of TAA used (43, 44).

Our analysis of allogeneic cancer vaccines addresses an additional concern—the off-target alloantigens presented by this platform represent a significant barrier to generating a tumor-specific immune response. When presented with thousands of foreign peptides, CD8^+^ T-cell responses are frequently directed toward a restricted group of or even a single antigen in a process known as immunodominance (45). While initially described in antiviral and antibacterial immune responses, this phenomenon and its implications are increasingly appreciated in vaccine administration (46, 47). Given that shared tumor antigens are outnumbered by off-target antigens in allogeneic vaccines by two orders of magnitude, it is unlikely that the predominant vaccine-induced response will be beneficial. Taken together, these data demonstrate the infeasibility of allogeneic vaccines as a source of immunologically relevant, potentially beneficial tumor antigens.

There are multiple limitations to this study that should be addressed. Regarding TAAs, our analysis was restricted to known TAAs, neglecting the possible contribution of unknown TAAs. In addition, TAA analysis was performed for the five commonest tumor types and did not evaluate the other 25 types included in the TCGA database. Finally, by only considering TAA peptides whose respective genes were expressed above the 50th percentile of all nonzero genes expressed in the tumor, we may discount the influence of TAAs less robustly expressed. The *in silico* nature of our analysis has several caveats. First, we used primary tumor sequence data from the TCGA to simulate the composition of allogeneic vaccines that would typically be made from cell lines. In addition, the samples chosen to simulate the allogeneic vaccine were randomly selected without optimization of TAA, neoantigen, or HLA coverage, which, if performed, would have the potential to increase the antigen repertoire of the vaccine. Similarly, restricting treatment to patients with certain HLA haplotypes would remove a potential barrier to immunogenicity and increase the number of patients capable of responding to vaccine antigens. Our methods rely heavily on the accuracy of neoantigen prediction software to provide the pool of possible overlapping neoantigens. While these are known to have flaws and differences exist between algorithms, the methods used here reflect those currently being used clinically (48–50). Despite the recent advances demonstrating the importance of the CD4^+^ T-cell response to tumor-specific mutations in the HLA-II context (51), we have not included HLA-II predictions in our models as they are much less reliable and have not begun full implementation in human trials as of yet (51, 52). Finally, the failure of allogeneic vaccines is likely multifactorial and not solely due to the paucity of beneficial antigens compared with distracting antigens. There are also many documented mechanisms of immunosuppression employed by cancer that likely play a role in the failure of vaccines (53).

Here we examine a shortcoming inherent to the design of allogeneic cancer vaccines. These data highlight the antigenic heterogeneity found within cancer types, the subsequent infeasibility of the allogeneic vaccine approach, and provide further support for a personalized approach to cancer vaccines. By evaluating individual tumors as opposed to relying on generic similarities within cancer types, personalized vaccines deliver neoantigens known to be expressed in the patient's tumor. Indeed, our analyses demonstrate the presence of numerous cancer neoantigens that can be targeted for each patient, with some melanoma patients exhibiting more than 200. The clinical relevance of these neoantigens can be seen in adoptive T-cell therapy, where they are being used to stimulate specific antitumor T cells, creating a more personalized adoptive T-cell therapy (e.g., NCT03171220 and NCT03658785). Indeed, in melanoma, neoantigen load was found to predict the clinical benefit of adoptive T-cell therapy (54). Identifying targetable neoantigens also facilitates personalized therapies in the form of chimeric antigen receptor T cells, capable of targeting neoantigens expressed solely on tumor cells (e.g., NCT02844062; ref. 55). Efforts are even being made to engineer TCRs against personalized neoantigens, treating patients with an infusion of these cells after expansion (e.g., NCT04102436). In addition, given the recent success of TAA vaccination in the proper context (56), personalized cancer vaccination allows for a hybrid approach, utilizing patient-specific neoantigens while also allowing for the inclusion of relevant TAAs. This approach has shown promise in phase I clinical trials in melanoma and there are ongoing trials determining their efficacy in a variety of cancers including pancreatic adenocarcinoma, breast cancer, and glioblastoma (57–60).

In summary, our results demonstrate that beneficial antigens provided by allogeneic vaccines are significantly outnumbered by off-target antigens, providing an immunologic hurdle to their clinical efficacy and potentially contributing to their failure in phase III trials. Given the rapidly decreasing cost, and increasing availability of next-generation sequencing, future efforts should be focused on pursuing personalized approaches with regards to cancer vaccines.

## Supplementary Material

Supplementary Data Table S1, Figures S1-S3
