# Supplementary material for: In Silico Epitope Prediction Analyses Highlight the Potential for Distracting Antigen Immunodominance with Allogeneic Cancer Vaccines
Source: Cancer Res Commun. 2021 Nov 30;1(2):115–26. doi: 10.1158/2767-9764.CRC-21-0029 (PMC9126504; doi:10.1158/2767-9764.CRC-21-0029)

Supplementary Tables/Figures

Table S1: Summary of Phase I/II Allogeneic Vaccine Clinical Trials

Cancer Type	Number of Trials (year began)
**Melanoma**	**28** (1985, 1985, 1988, 1991, 1992, 1994, 1997, 1998, 2001, 2002, 2002, 2002, 2002, 2002, 2004, 2004, 2005, 2006, 2006, 2008, 2008, 2009, 2009, 2011, 2011, 2013, 2013, 2020)
**Prostate**	**16** (1995, 1998, 1999, 1999, 2001, 2004, 2004, 2005, 2005, 2005, 2006, 2007, 2008, 2008, 2009, 2013)
**Pancreas**	**15** (1996, 2001, 2002, 2005, 2005, 2006, 2008, 2008, 2008, 2009, 2010, 2013, 2015, 2016, 2016)
**Lung**	**14** (2003, 2003, 2003, 2006, 2006, 2006, 2006, 2009, 2010, 2011, 2013, 2013, 2015, 2018)
**Breast**	**11** (1993, 1996, 2002, 2004, 2004, 2004, 2006, 2008, 2008, 2009, 2018)
**Colon**	**6** (1991, 2000, 2008, 2008, 2013, 2019)
**Neuroblastoma**	**5** (1991, 1998, 1998, 2008, 2010)
**Glioblastoma**	**3** (2013, 2014, 2019)
**Renal**	**2** (2010, 2011)
**Myeloma**	**1** (2017)
**Multiple**	**1** (2017)
**Mesothelioma**	**1** (2017)
**CML**	**1** (2004)

Figure S1

Allogeneic cancer cell vaccine construction: (**A)** (1) A tumor is excised and an immortalized cell line is established in vitro or a previously established cell line is identified and expanded. (2)The cell line may be modified to improve immunogenicity. (3) The cell line is lethally irradiated to prevent ongoing cell division/multiplication within patient once vaccinated. (4) Vaccine is delivered by injection into either muscle or tumor site, depending on anatomic accessibility. (**B**) **Whole Cell vs Cell-Lysate Vaccination**: When vaccinating with whole cells, allogeneic tumor cells are responsible for presenting tumor antigens to host antigen presenting cells (APC). Consequently, MHC-matching is required for immunogenicity. This is in contrast with the lysate vaccination strategy, where host APCs take up cellular debris and tumor antigens are obtained directly.

1A

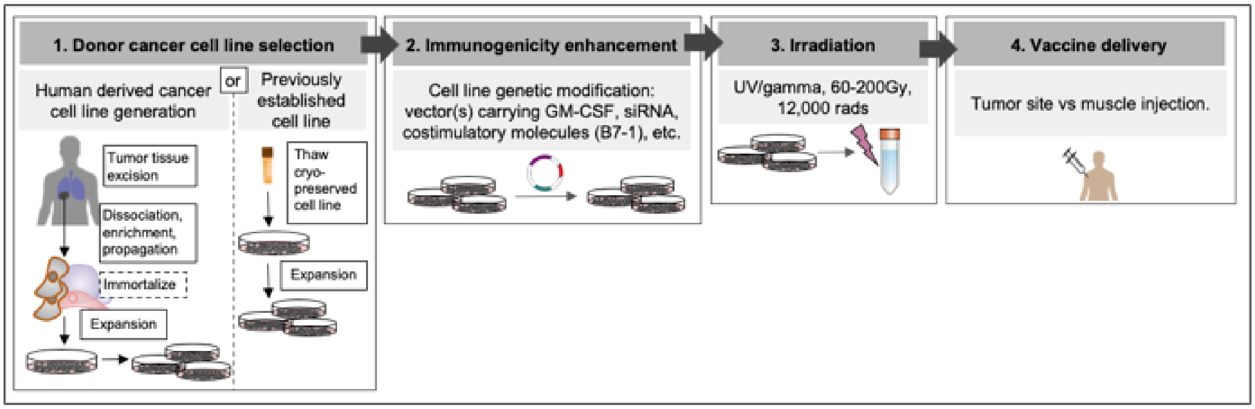

1B

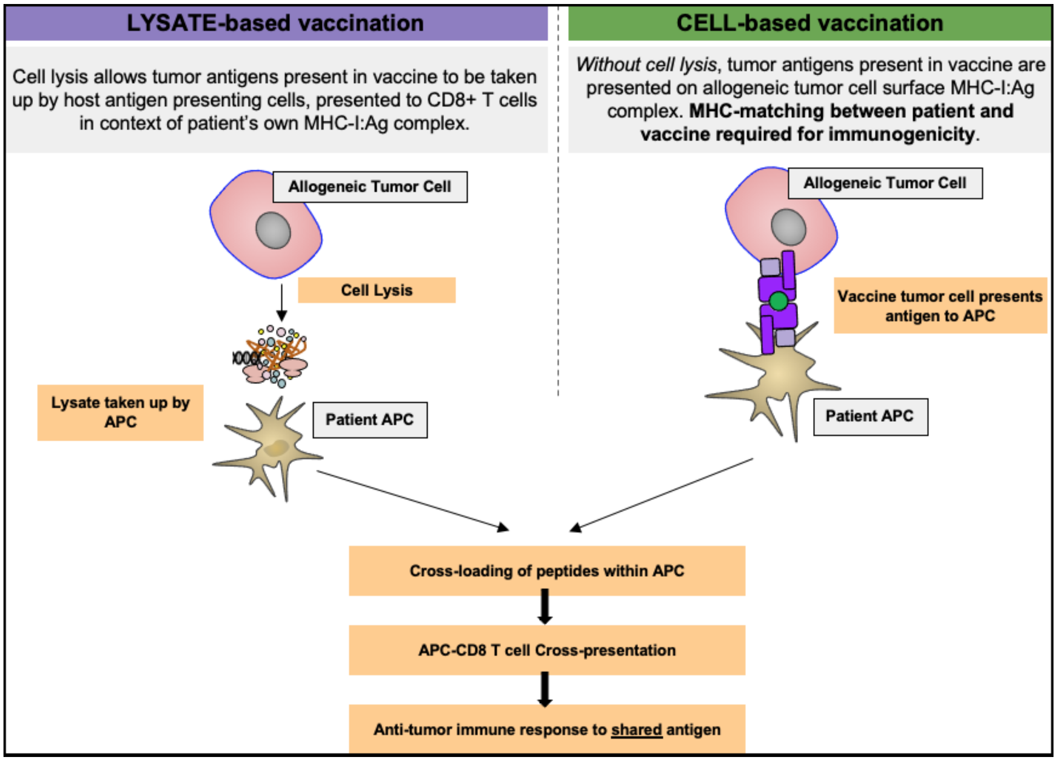

Figure S2

Pan cancer lysate-based neoantigen analysis of shared neoantigens

(**A**) Counts of shared neoantigens per patient for each cancer type based on a lysate-based vaccine administration. Counts summarize 10 complete trial simulations (300 simulated patients total) for each of the 30 TCGA Cancer types. (**B**) A heatmap of shared neoantigen identities from combined trials in each cancer type (300 simulated patients).

A

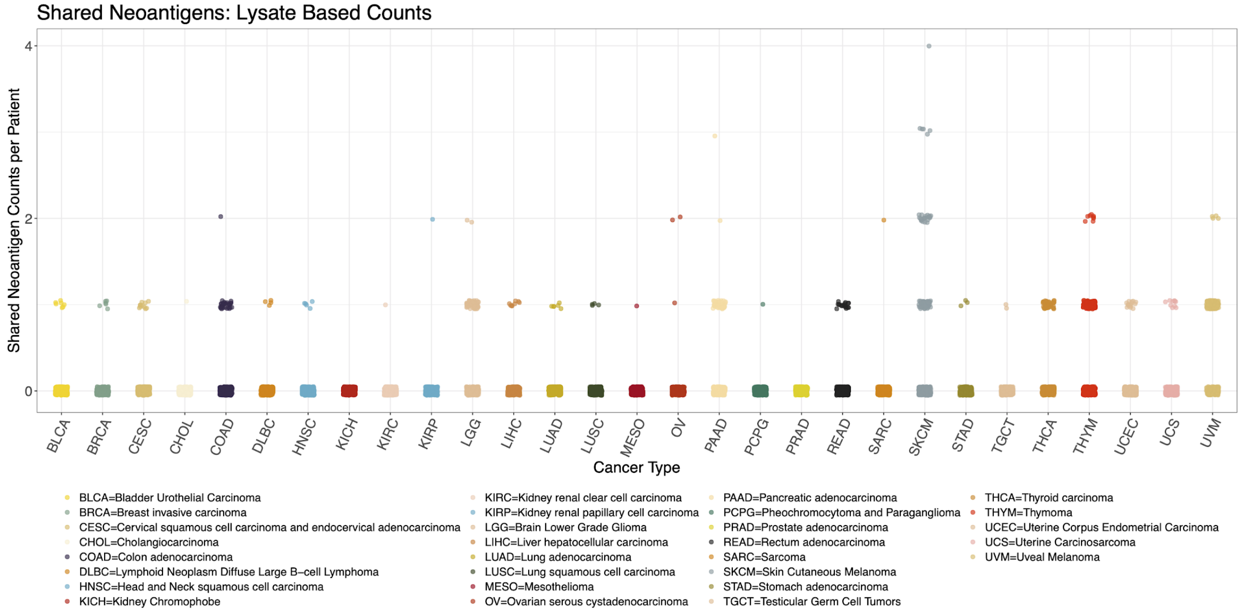

B

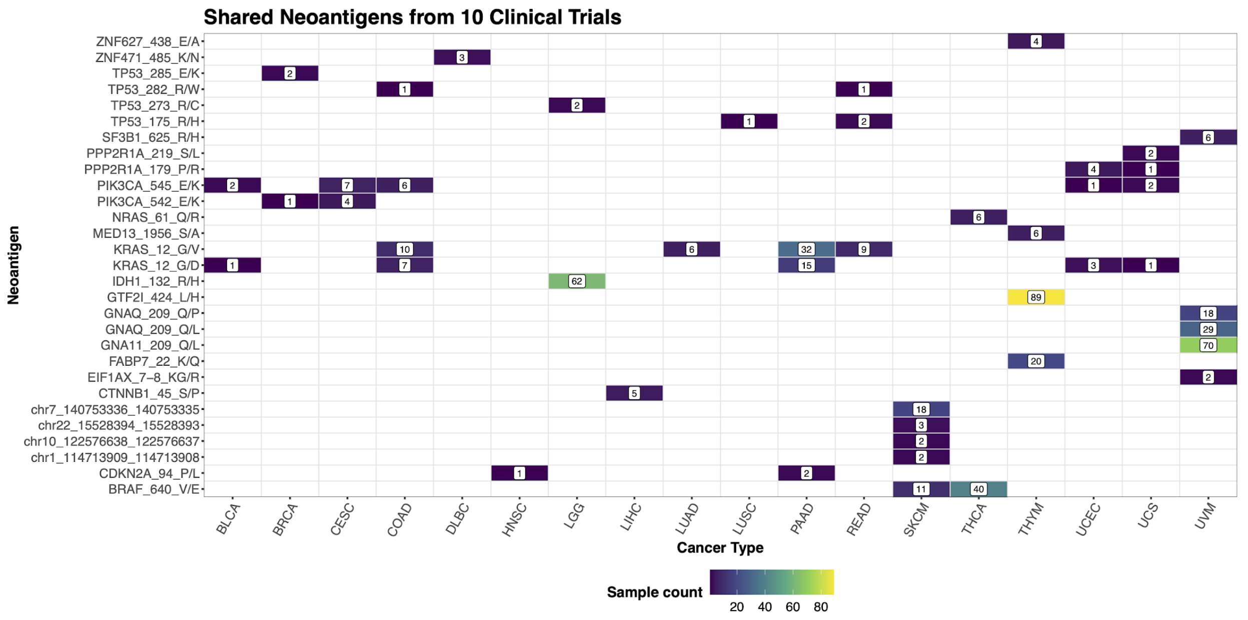

Figure S3

Pan cancer cell-based neoantigen analysis of shared neoantigens

(**A**) Counts of shared neoantigens per patient for each cancer type based on a cell-based vaccine administration. Counts summarize 10 complete trial simulations (300 simulated patients total) for each of the 30 TCGA Cancer types. (**B**) A heatmap of shared neoantigen identities from combined trials in each cancer type (300 simulated patients).

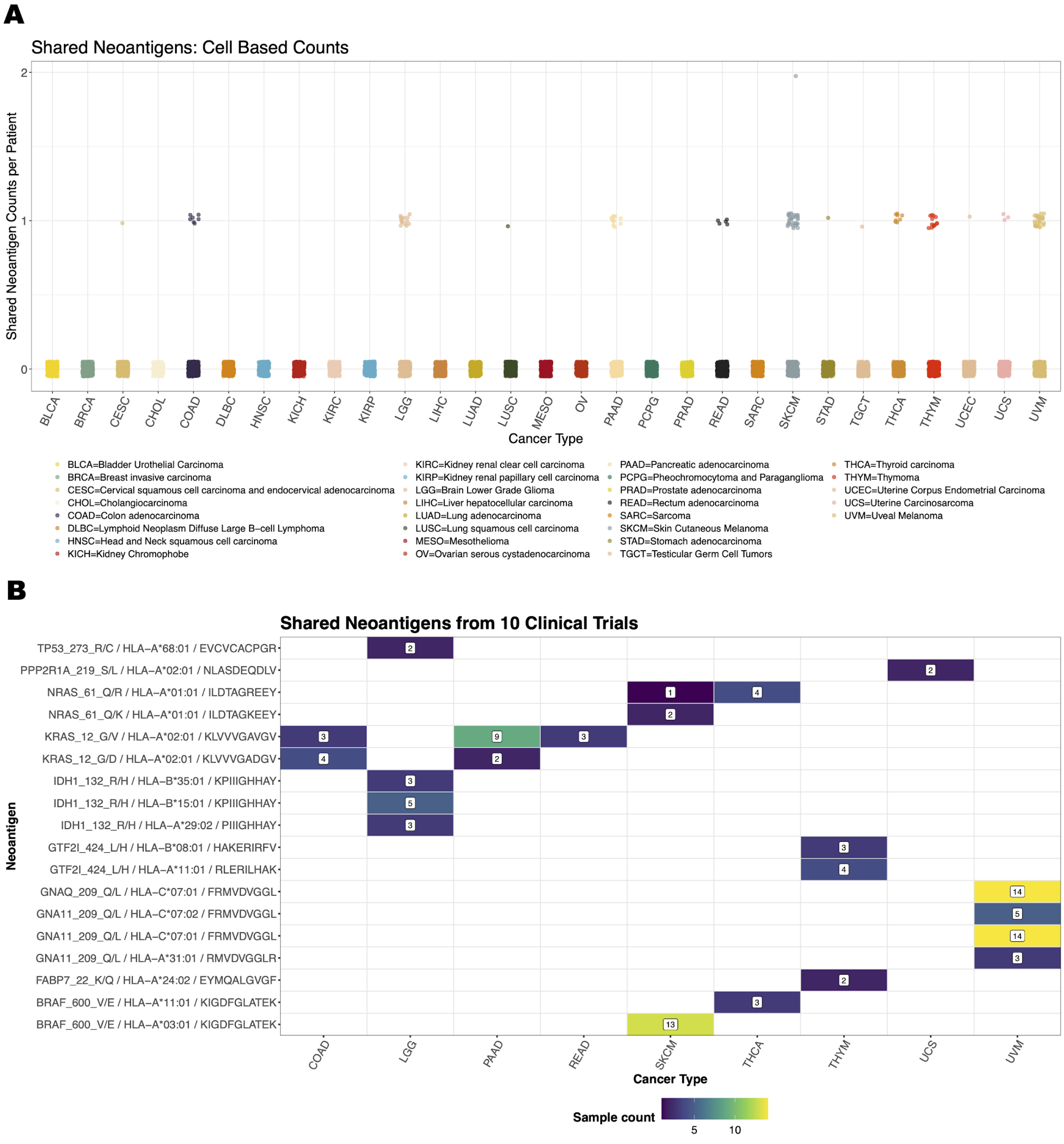

Supplement: Supplementary Data — Table S1, Figures S1-S3 [file crc-21-0029-s01.pdf]
